# Direct Silver Micro Circuit Patterning on Transparent Polyethylene Terephthalate Film Using Laser-Induced Photothermochemical Synthesis

**DOI:** 10.3390/mi8020052

**Published:** 2017-02-13

**Authors:** Chen-Jui Lan, Song-Ling Tsai, Ming-Tsang Lee

**Affiliations:** Department of Mechanical Engineering, National Chung Hsing University, Taichung 402, Taiwan; lan.andy@gmail.com (C.-J.L.); s3677265@gmail.com (S.-L.T.)

**Keywords:** laser direct synthesis and patterning, flexible electronics, laser direct write, transparent conductors

## Abstract

This study presents a new and improved approach to the rapid and green fabrication of highly conductive microscale silver structures on low-cost transparent polyethylene terephthalate (PET) flexible substrate. In this new laser direct synthesis and pattering (LDSP) process, silver microstructures are simultaneously synthesized and laid down in a predetermined pattern using a low power continuous wave (CW) laser. The silver ion processing solution, which is transparent and reactive, contains a red azo dye as the absorbing material. The silver pattern is formed by photothermochemical reduction of the silver ions induced by the focused CW laser beam. In this improved LDSP process, the non-toxic additive in the transparent ionic solution absorbs energy from a low cost CW visible laser without the need for the introduction of any hazardous chemical process. Tests were carried out to determine the durability of the conductive patterns, and numerical analyses of the thermal and fluid transport were performed to investigate the morphology of the deposited patterns. This technology is an advanced method for preparing micro-scale circuitry on an inexpensive, flexible, and transparent polymer substrate that is fast, environmentally benign, and shows potential for Roll-to-Roll manufacture.

## 1. Introduction

The market and applications of flexible, stretchable, and wearable electronics have increased remarkably in recent years. The search for rapid and cost-effective ways to combine electronics with flexible and stretchable substrates has also grown enormously. One central issue that challenges the fabrication of flexible electronics is that most flexible substrates, such as polymers, are unsuitable for the conventional photolithography used in microelectronic fabrication because they have poor chemical and thermal resistance. The thermal issue also leads to poor adhesion between the metallic materials and the polymer substrate caused by significantly large differences in thermal and mechanical properties [1]. To overcome these problems, especially the thermally related limitations, several advanced low-temperature manufacturing techniques have been developed that allow high resolution patterning [2]. These include nano-imprinting [3], transfer printing [4], precision deposition and low temperature process using nanoparticle ink [5], and laser-assisted direct sintering and patterning of nanomaterials [6,7,8]. These techniques take advantage of the melting point depression effect of nanomaterials, which allows the nanoparticles of metal to be easily sintered at moderate temperature [9]. The material is deposited on the substrate from a solution followed by low temperature sintering to form highly conductive nano-/microscale structures. The pattern can be produced by selective deposition or selectively sintering. The nanomaterial-based fabrication technique is fast, maskless, and gives reasonably good resolution mainly depending on the laser parameters and optics. Large area processing for Roll-to-Roll manufacturing is also possible by the laser-nanomaterial-based microfabrication technique. 

However, all these nanomaterial-based processes require the synthesis of metal nanomaterials, which is usually a complex, time-consuming, and costly procedure. The coating and recycling of nanomaterial on a polymer substrate is also very challenging. The novel laser direct synthesis and pattering (LDSP) technology presented here provides a low temperature, green, nanoparticle-free process that does not require masks, or vacuum, and is ideal for the rapid fabrication of flexible electronics [10,11]. In contrast to the nanomaterial-based laser-assisted manufacturing processes such as laser direct write that utilizes a pre-synthesized metal nanomaterial solution to deposit the material followed by laser sintering, a transparent and particle-free metallic ion reaction solution is applied to the polymer substrate in the LDSP process. A focused continuous wave (CW) laser beam is scanned across the polymer surface to create a pattern. The laser energy absorbed by the tinted polymer substrate heats the reaction solution near the interface. Metallic ions are reacted and precipitate to form the predetermined pattern on the substrate surface. LDSP uses a nanoparticle-free metal ion precursor—not metal nanoparticle ink—which makes the process significantly faster. The process is simpler and green when compared to conventional laser direct sintering of metal nanoparticles. However, one limitation of the LDSP process is that the polymer substrate needs to absorb the scanned laser light. In other words, if an economic CW visible laser is to be used as a heat source, then the polymer substrate has to be tinted. However, for applications needing highly transparent and flexible electrodes, such as touch panels and flexible solar cells, a transparent polymer substrate has to be used. In such a case, the reactive ionic solution itself must absorb the incident laser light. A possible solution is to pre-deposit an absorbing layer on the substrate [12]. However, this could induce the complexity of the fabrication process and could also affect the transparency of the substrate. Another straightforward solution involves mixing the transparent ionic solution with a highly absorptive dye. The laser beam can then be directed through the transparent substrate to the top surface from below where it will be absorbed by the adjacent solution to initiate a thermochemical reaction and cause silver synthesis and patterning.

Although conceptually promising, the feasibility and the consequences of this approach remain to be proved. In addition, for realization of economic flexible electronics, low-cost transparent substrates such as polyethylene terephthalate (PET) film should be used. However, this kind of substrate is usually vulnerable to elevated temperature during the fabrication process. The thermal impact on the PET substrate subjected to the modified LDSP process also needs to be investigated. In this study, an improved LDSP process has been modified as described. Specifically, a red dye was added to the transparent nanoparticle-free reactive ion solution. The solution was applied to the transparent PET substrate, after which the LDSP process, where a focused laser beam was directed through the underside of the transparent PET substrate, was carried out. The red-colored reactive solution serves as the absorber of photon energy and converts it to thermal energy for the chemical reaction. The effects of the additive materials, concentration and the number of laser scans were all investigated. Electrical conductivities and the mechanical durability of the resulting nano-/microscale conductive patterns on the transparent PET substrate were measured and discussed. It is emphasized that, and shown in the results, using the red dye to make the reactive solution absorptive is a simple and cost-effective way to expand the application of the LDSP technology to include transparent polymer substrates. Unlike the other possible types of additives where nano-powders are used to form adsorptive colloids, the reactive solution mixed with red dye used in this study is free from precipitation and unwanted chemical reactions caused by the use of additives. Numerical simulation was also carried out on the heat and flow transport near the laser focal spot. Discussions of the impact on the resulting silver line structures from the temperature and velocity fields are also provided.

## 2. Materials and Methods

### 2.1. Processing Solution Preparation

The transparent reactive silver ion solution was prepared following a reported procedure [13] with modification. In brief, 1.0 g of silver acetate (anhydrous 99%, Alfa Aesar, Ward Hill, MA, USA) was dissolved in 2.5 mL of aqueous ammonium hydroxide (28%~30%, ACS Reagent, J.T. Baker, Phillipsburg, NJ, USA) at room temperature with vigorous stirring, followed by the drop-wise addition of 0.2 mL of formic acid (88%, ACS Reagent, J.T. Baker). The solution was filtered through a 220 nm poly(vinylidene fluoride) (PVDF) filter. The resulting particle-free clear ionic solution contains approximately 22% silver. Separately, a red dye, Allura red AC, was dissolved in ethylene glycol (EG, 99%, Alfa Aesar) under sonication to give solutions with molarities of 8 mM, 16 mM, and 32 mM. The red-EG was then mixed with the ionic solution at a volumetric ratio of 1:1 to complete the preparation of the EG–silver ion reaction solutions. Consequently, process solutions with red-dye concentration of 4 mM, 8 mM, and 16 mM were utilized in the current study. It should be pointed out that the red dye was chosen in the current study owing to its effective absorption to the green light laser energy, as also to be shown and discussed in the following section regarding the optical property of the red dye.

### 2.2. The LDSP Process

**Figure 1:**
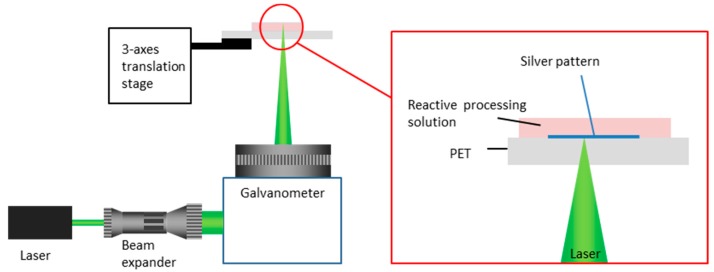
Experimental apparatus for the modified laser direct synthesis and pattering (LDSP) process.

**Figure 2:**
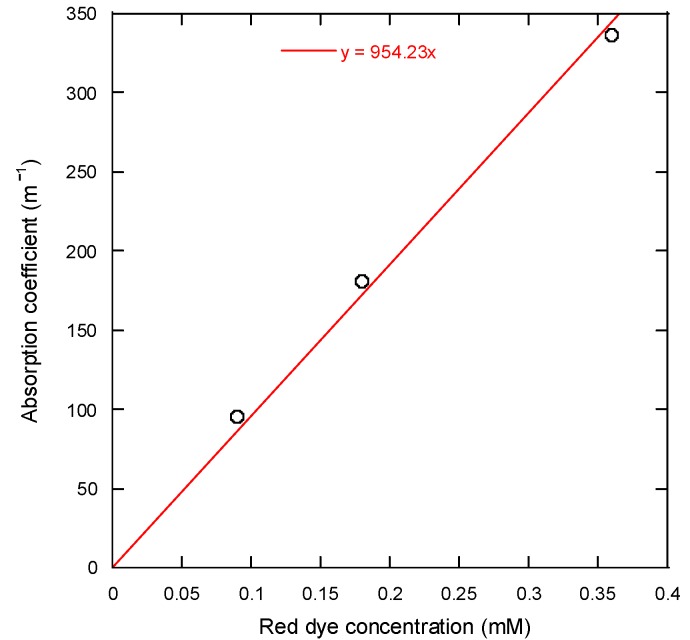
The absorption coefficient with respect to the red dye concentrations in the process (Ag–EG) solution.

The diagram in Figure 1 illustrates the setup of the experimental apparatus used in the LDSP process. The 50-µm-thick PET film with an area of 5 cm × 5 cm used for this experiment was cleaned by rinsing with ethanol and DI water consecutively. The film was then treated with oxygen plasma in an Atmospheric Pressure Plasma Cleaner (Output voltage: 10,000 V to 50,000 V, output frequency: 4 MHz to 5 MHz) for 10 s to make the surface hydrophilic and improve adhesion of the silver to the polymer surface. The PET substrate was then placed carefully on a glass slide sample holder. The process solution was pipetted onto the substrate surface to form a liquid film typically 2~3 mm thick. The thickness of the process solution was controlled by controlling the amount of the solution being dispensed. It should be noted that the LDSP process may be sensitive to liquid film thickness as discussed in our previous studies [10,14]. As also to be shown from the numerical simulation results, the range of temperature and fluid flow in the process solution affected by the focused laser is confined within 1 mm surround the focal point. Therefore, 2~3-mm-thick process solution in the current study is sufficient to prevent the impact from the liquid-air boundary. The laser beam from a continuous wave (CW) diode laser (λ = 532 nm) was aligned and focused to 50 μm in diameter on the top surface of the PET substrate from underneath with the assistance of a beam expander and beam profile analyzer (BeamGage^®^ Beam Profiler SP620U, Ophir Optronics^®^, Jerusalem, Israel). The focused laser beam was then directed by a programmable galvanometer scanner system (SCANLAB hurryScan^®^ II-7, SCANLAB GmbH, Munich, Germany) to selectively scan a predetermined light pattern through the transparent PET substrate, the energy of which was absorbed by the process solution. Since the PET substrate is transparent and thin, there was no significant extinction of the laser light and the process solution absorbed the laser energy very effectively. Figure 2 shows the absorption coefficient of the process solutions with respect to the concentration of the red dye. Measurements were carried out using a UV-Vis spectrophotometer (Genesys 10S, Thermo Scientific, Waltham, MA, USA). Due to the limited measurement range of the spectrophotometer, three diluted solution concentrations were used. Assuming negligible scattering and from extrapolation of the measured absorption coefficient using the fitted curve as shown in Figure 2, the penetration distance attributed to absorption by the 16 mM process solution was approximately 65 μm [15]. This estimated result suggests that the absorption of the incident laser energy, and consequent heating of the process solution, would be confined to a vicinity of ~O(10^2^ μm) to the PET surface. A small and well controlled thermal impact area is essential for the LDSP process to ensure good resolution as well as the recovery of unreacted process solution. After the LDSP process was completed, the PET surface was washed carefully with deionized water and ethanol and air-dried to remove any remaining process solution.

## 3. Results

**Figure 3:**
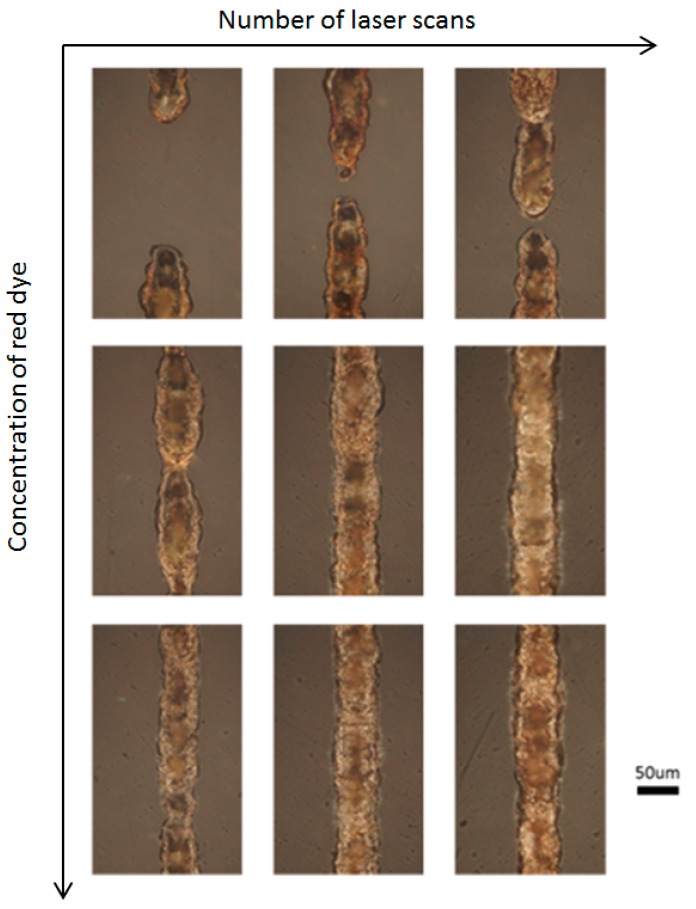
Silver lines fabricated by the LDSP process on the polyethylene terephthalate (PET) substrate with different numbers of laser scans: 10×, 20×, 30× and red-dye concentrations in the process solution of 4 mM, 8 mM, and 16 mM.

**Figure 4:**
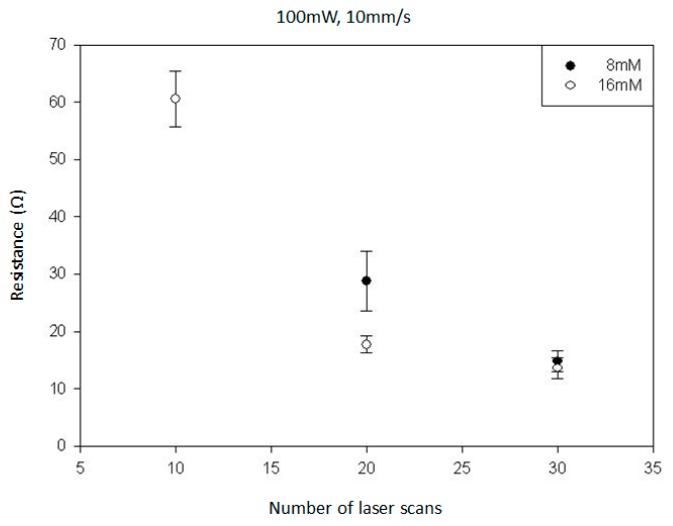
Electrical resistivity of silver lines with respect to the red-dye concentration and number of laser scans.

In this study, the laser power was fixed at 100 mW, and the laser scan speed was 10 mm/s. The number of laser scans and the red-dye concentrations were varied to conduct the parametric studies. Figure 3 shows the silver lines fabricated by the LDSP process on PET substrate with a range of different laser scans (10×, 20×, 30×) and red-dye concentrations (4 mM, 8 mM, 16 mM, based on the mixed process solution). It can be clearly seen that the 4 mM red-dye process solution did not yield a continuous silver line even when the maximum number of scans (30×) was applied. The formation of such broken lines also indicates that the thermochemical reduction of silver ions on the PET surface is not uniform and steady. The temperature and the reactant concentration are two major factors affecting the chemical reaction rate and it is possible that the concentration of reactants and the red dye was not uniform along the laser scan path. In these experiments, the red dye in the reactive solution absorbs laser energy and participates in radiative heat transfer. The reaction temperature and thermally induced convection is affected by the absorption of the red dye in the solution and although the process solution appears to be homogenous, there could still be microscale fluctuations in concentration. Thermally induced fluid motion such as buoyancy-driven flow, the Soret effect, and Brownian motion will also affect the concentration of species in the solution [16]. These fluctuations are highly random, especially for solutions of low red-dye concentration, and increasing the number of laser scans can build the line incrementally. Nevertheless, for a real application, the number of scans should be minimal and the red-dye concentration was increased to improve uniformity and the absorption of laser light. As can be seen in Figure 3, the silver lines fabricated using a red-dye concentration of 8 mM yields continuous conductive lines after 20 laser scans and 10 laser scans were enough to give continuous lines with a 16 mM solution. The width of the deposited line is approximately 50 μm. The size and the resolution of the conductive patterns being fabricated by the LDSP process is mainly controlled by the size of the heat affect area [14], which are determined by the process parameters such as the laser intensity, scanning speed, and heat transfer properties of the process solution. In addition, the size and morphology of the LDSP deposited patterns could also be controlled with the assistance of microfluidic dispensers [17] in conjunction with adjusted laser parameters. Figure 4 shows the electrical resistivity, where measurable, of these silver lines. The resistivity decreased to become stable with an increase in the number of laser scans or red-dye concentration. It should be noted that, for the 16 mM solution, silver lines with 20 laser scans and 30 laser scans yield compatible resistivity, which suggests that the effective thickness of the line did not increase significantly with more than 20 laser scans. A similar trend had also been observed earlier [10,14]. This effect is attributed to the fact that, as the thickness of the silver line increases, it begins to reflect the incident laser light, the energy is no longer absorbed by the process liquid, and the chemical reaction therefore stops.

**Figure 5:**
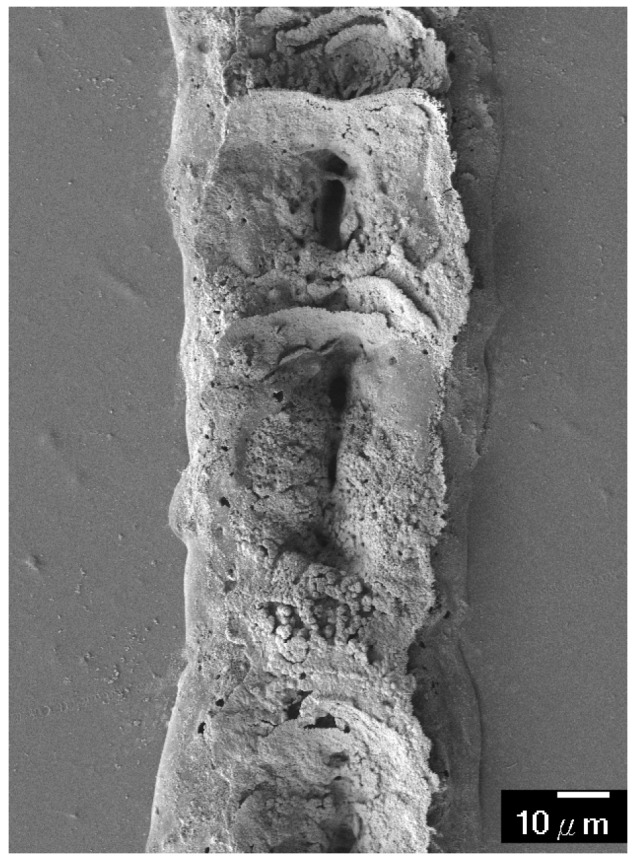
Scanning electron microscope (SEM) image of a silver line fabricated using a 16 mM process solution and 30 laser scans.

Figure 5 shows the silver line fabricated with the 16 mM process solution and 30 laser scans. The rough surface mainly results from solution flow and the possible sintering of silver nanoparticles that were produced during the laser scan [18], which could be improved by increasing the viscosity of the fluid [10]. The cross section of the silver line exhibits a concave shape. This surface morphology is one of the typical shapes found for patterns fabricated by laser chemical vapor deposition (LCVD), which is similar to the mechanism of LDSP. The formation of the morphology is closely related to the temperature distribution, the transport of reactants and products in the liquid (in this case, possibly silver nanoparticles being produced during the laser scan) and the surface diffusion of species [19]. The concave and round shape indicates strong impact from capillary-driven flow and thermophoresis, which are significant when the local temperature gradient in the fluid near the laser focal spot is large.

**Figure 6:**
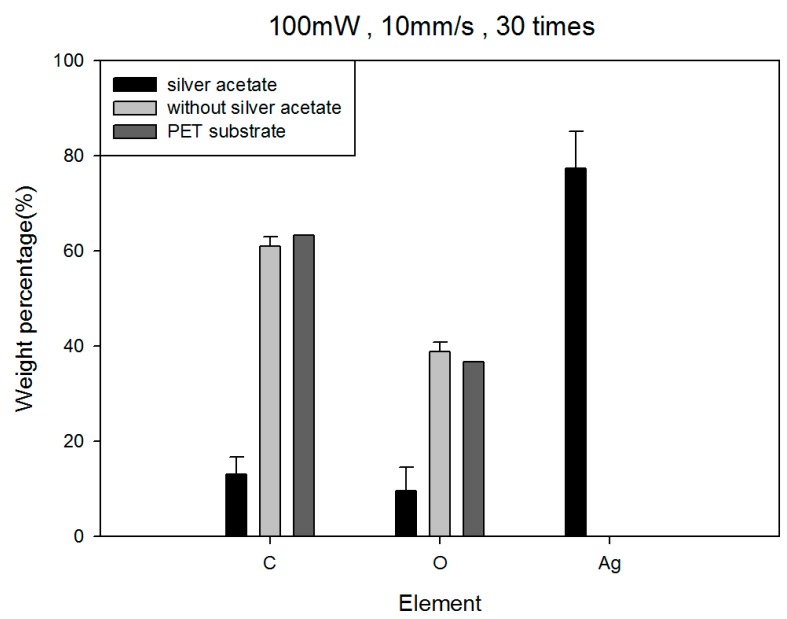
Energy dispersive X-Ray analysis (EDX) elemental analyses.

Figure 6 shows the results of energy dispersive X-ray analysis (EDX) analysis. It can be seen that the PET substrate after the LDSP in the process solution without silver acetate (but with red dye) contains no silver element, and the ratio of carbon to oxygen slightly increased compared with the original PET substrate. It can be assumed that very little red dye would be sintered and deposited on the PET substrate during the LDSP process. Silver lines fabricated with the process solution containing silver acetate shows dominant silver content and a small amount of carbon and oxygen. The carbon and oxygen could be from the red dye embedded in the silver line. These carbon and oxygen components in the conductive pattern are thought to be the main reason for its higher resistivity compared to pure silver.

**Figure 7:**
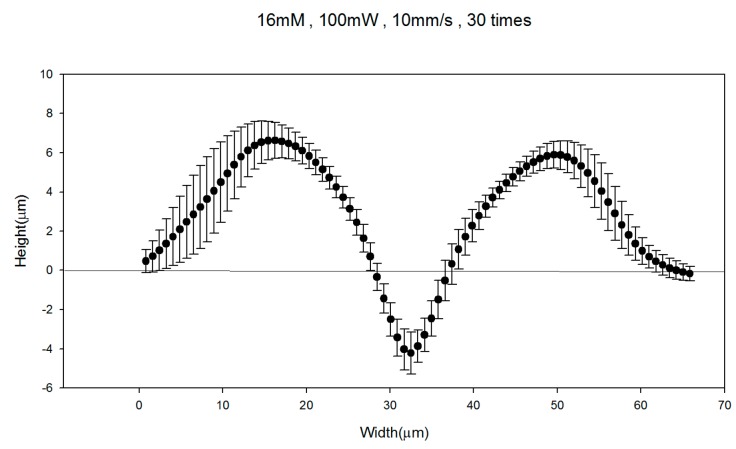
The morphology of the cross section of a silver line fabricated with LDSP (16 mM process solution, 30 laser scans).

**Figure 8:**
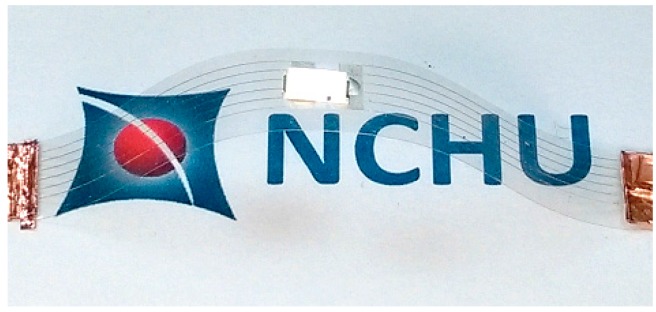
Demonstration of conductive silver line fabricated by LDSP on the transparent PET substrate.

**Figure 9:**
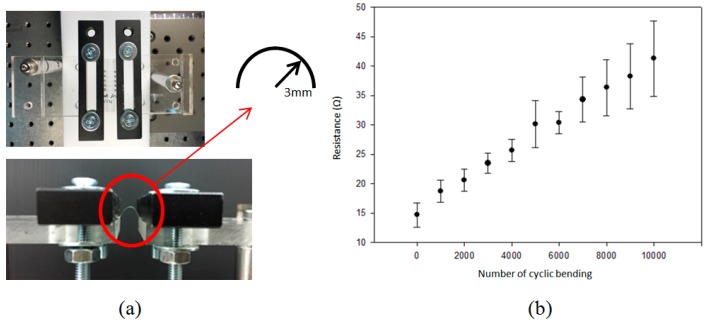
(**a**) Bending test apparatus; (**b**) resistance drift.

Figure 7 shows the morphology of the cross section of the silver lines fabricated with LDSP (16 mM process solution, 30 laser scans). The measurements were carried out using a white light interferometer. The concave morphology can be clearly seen. The negative value at the center of the silver line could be attributed to slight melting of the PET substrate during the LDSP process. Using the average altitude profile shown in Figure 7 to calculate the cross-sectional area of the line, and assuming the canyon at the center under the plain substrate surface does not contribute to significant electrical conductivity, the electrical resistivity of the silver line was estimated to be 2.25 × 10^−7^ Ω·m, which is approximately 10 times that of pure silver. This can again be attributed to the fact that the composition of the conductive line contains a fraction of carbon and oxygen. Nevertheless, the conductivity of the line is sufficient to conduct electricity effectively as demonstrated in Figure 8. The durability test apparatus and the results are shown in Figure 9. After 10,000 repetitive bends, the drift in electrical resistance of the silver line was approximately 3 times higher than the initial value. Referring to the microstructures of the silver line showing in Figure 9b, there are silver micro-flakes that tend to be easily deformed and deteriorated. The variation in the electrical property is mainly attributed to the change in mechanical structure of the line. Further effort should be made to improve the mechanical robustness and the durability of the conductive patterns fabricated by this technique. It should also be noted that the processing speed could be increased by increasing the laser intensity, the absorptivity, and/or the reactivity of the process solution. The productivity of the LDSP process can thus be improved.

Numerical simulations were carried out to further investigate the transport phenomena during the fabrication process. COMSOL Multiphysics^®^ software (COMSOL Inc., Burlington, MA, USA) was applied in the current study. Details of the numerical model can be found in an earlier report [14] and in [19,20]. In comparison to our previous numerical study of transport phenomena in the LDSP process, the main difference in the current numerical model is the absorbing material. In earlier studies, the substrate (polyimide film) served as a laser light absorbing material, while in the current study the laser energy was absorbed by the process solution. The heat source *Q*_abs_ from the absorption of a Gaussian laser beam moving in the liquid can be determined by [3,20,21]:

(1) $$Q_{\mathrm{abs}}=\left(1-R\right)\gamma I_{\mathrm{pk}}\exp\left[-{\left(\frac{x-Ut}{\omega }\right)}^{2}-{\left(\frac{y}{\omega }\right)}^{2}-\gamma z\right]$$

where *R* is the reflectivity, γ is the absorption coefficient of the process solution estimated from Figure 2, *I*_pk_ is the peak laser intensity, and *ω* is determined from the size of the laser beam on the *x*-*y* plane of the PET substrate. In the current simulation, the surface reflectivity from the PET substrate and the interfaces of the PET and process solution were assumed to be negligible. Chemical reactions and liquid-vapor phase change were also neglected in this analysis considering that there was no significant boiling observed during the process.

**Figure 10:**
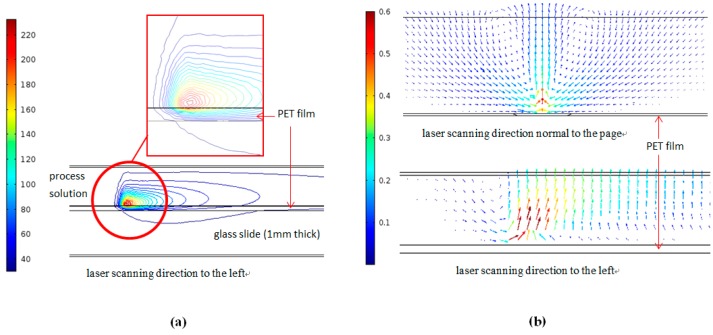
(**a**) Temperature (unit: °C) profile; and (**b**) velocity (unit: mm/s) profile near the laser focal point.

Figure 10a shows the temperature profile of the cross section at the center of the laser beam in the scan direction. It can be seen that the highest temperature spot is inside the reaction fluid and is some tens of microns above the PET surface. In contrast to the previous studies where the laser beam energy was absorbed by the polymer substrate itself, so that the highest temperature in the reaction fluid was on the interface between the solid substrate and the fluid, the process solution absorbs the incident laser energy gradually along the laser path within the fluid, and the hottest spot is in the interior of the process fluid region. This, in turn, results in a strong in-plane flow right on the PET surface below the hottest spot. Strong in-plane flow, especially near the centerline (the symmetric line), could adversely affect the deposition and adhesion of the reduced silver (from the hottest spot) on the PET surface. Additionally, the symmetric swirling flow as shown in Figure 10b indicates that the reduced silver could precipitate on the shoulders along the laser scanning path, which also corresponds to the morphology of the silver line patterns shown in Figure 5. It should also be noted that the upward flow at the centerline due to buoyancy exists with an absorptive substrate (polyimide) or an absorptive fluid as in the current case. The morphology of the deposited pattern, however, is closely related to the intensity of the buoyancy flow, which generally increases with laser intensity, which determines the temperature of the focal point [19]. Other forces such as surface tension would be important for improving the surface morphology [22]. Furthermore, the roughness of the surface of the deposited patterns could yield a random scattering of the incident laser irradiation [23]. Thus, the reflectance to the laser light by the deposited patterns should be carefully considered. Continuous study in this regard is being carried out.

## 4. Conclusions

In the current study, a new and improved approach to the rapid and green fabrication of highly conductive nano-/micro-scale silver structures on low-cost transparent polyethylene terephthalate (PET) flexible substrate was successfully demonstrated. Predetermined silver pattern was formed by photothermochemical reduction of the silver ions induced by the focused CW laser beam in a processing solution containing a red azo dye as the absorbing material. The effects of the additive materials, concentration, and the number of laser scans were all investigated. The electrical resistivity of the silver line was estimated to be 2.25 × 10^−7^ Ω·m, which is approximately 10 times that of pure silver. Numerical simulation was also carried out on the heat and flow transport near the laser focal spot to provide insights of the impact on the resulting silver line structures from the temperature and velocity fields. The concave and round shape of the conductive lines indicates strong impact from capillary-driven flow, thermophoresis, and buoyancy flow in the current LDSP configuration. This study and the revised LDSP technology provides a potential approach for preparing nano/micro-scale circuitry on inexpensive, flexible, and transparent polymer substrates that are fast, environmentally benign, and cost-effective.
